# Word of mouth from an adolescent with cancer in the convalescence stage to exploring their mental health after treatment

**DOI:** 10.1038/s41598-024-51836-8

**Published:** 2024-01-16

**Authors:** Lu Yu, Chunhai Gao, Xinyu Zhang, Sabika Khalid, Endale Tadesse, Lin Mo

**Affiliations:** 1 Department of Outpatient, Children’s Hospital of Chongqing Medical University, 136 Zhongshaner Rd, Yuzhong District, Chongqing, 400015 China; 2 National Clinical Research Center for Child Health and Disorders, Chongqing, China; 3 Ministry of Education Key Laboratory of Child Development and Disorders, Chongqing, China; 4 China International Science and Technology Cooperation Base of Child Development and Critical Disorders, Chongqing, China; 5 Chongqing Key Laboratory of Pediatrics, Chongqing, China; 6 Faculty of Education, Shenzhen University, Shenzhen, Guangdong China; 7 School of Clinical Medicine, The Zhuhai Campus of the Zunvi Medical University, Zhuhai, China; 8 College of Teacher Education, Zhejiang Normal University, Jinhua, Zhejiang China

**Keywords:** Psychology, Health care

## Abstract

To explore the psychological experience and emotional needs of returning to family and society after treatment in an adolescent with cancer. A phenomenological research design was employed to conduct a semi-structured interview with nine cancer adolescents in the convalescence stage. Colaizzi’s seven-step analysis was used for the data analysis. Three themes of the psychological experience of an adolescent with cancer in convalescence were summarized: continuous negative emotions, emotionally intense behavior, and discomfort of returning to society. The psychological experience of an adolescent with cancer in convalescence returning to family and community is sensitive and complex. Medical staff, families, and schools should provide personalized care according to their different psychological characteristics and emotional needs so that they can return to family and society smoothly.

## Introduction

Cancer is the fourth leading cause of death worldwide among adolescents and young people^1^. Every year, about 105,000 adolescent cancer patients are added worldwide^2^, with a rising rate of 1.5% per year^3^. Though with advancements in the field of medicine, the survival rate of cancer patients surpasses 80%^4^, the cancer convalescence stage, which is the process of recovery from the disease, left significant effects on adolescent physical and psychological health even after the cancer treatment^5^. The most severe impact is Cancer-related cognitive impairment (CRCI), which significantly affects the quality of life of survivors^6^. CRCI condition affects a person’s thinking, reasoning, and learning processes^7^. Due to the CRCI condition, the total recovery time takes way longer than the actual treatment of cancer disease^8–10^. Cancer patient survivors are also faced with severe psychological distress^11^, which affects their emotional well and personal lifestyle and leads to financial problems in their lives^12^.

It is estimated that from 2018 to 2020, 121,145 children and adolescents were diagnosed with cancer in China^13^, and the incidence rate among adolescents was 137.64/million. Adolescents with cancer are a particular group of cancer patients. On the one hand, adolescence is a crucial period for forming an outlook on life and values. This period is characterized by a desire for independence, rapid physical and mental development, and full of contradictions. On the other hand, cancer has changed adolescents’ lifestyles, affecting their physiology, psychology, education, social interaction, and other aspects. In addition, adolescent patients’ coping styles and psychological status differ from those of adults. So, the focus of care should also be different.

Investigating the young adult population cancer in convalescence is very important as their psychological health can seriously disrupt their abilities to establish functional roles in society^14^. Literature on this area of psychological health after cancer treatment is mainly related to middle-aged and older breast cancer survivors^7^; the young adults’ experiences during cancer treatment remain poorly understood^15^. Another limitation of previous studies is that most studies were quantitative and failed to provide young adults’ lived experiences with this adverse effect^16^.

Currently, there are some qualitative studies internationally on the psychological experience of adolescents with cancer during the diagnosis or treatment period^17,18^. However, the qualitative literature on this aspect is very limited in China, where the ratio of cancer in adolescents is relatively high^4^. As a large population country, China is one of the countries facing the immense responsibility of childhood cancer^16^. The present study aimed to deeply research the psychological course and emotional needs of adolescents with cancer in the convalescence stage through phenomenological research. The results provide ample information to medical personnel and the government community to formulate personalized and targeted interventions.

Thus, this study aims to explore adolescents’ psychological challenges and concerns during the cancer convalescence stage and their mental well-being in living in society after the treatment.

## Materials and methods

### Design

A phenomenological research design was used to carry out the research. The existential phenomenology approach was used to understand the participant’s experiences through their perspective^29^. The study is approved by the research ethics committee of Chongqing Medical University, which is conducted in accordance with the Declaration of Helsinki’s guidelines and regulations.

### Participants selection

Purposive sampling was used to ensure the sample was broadly diverse. The study used specific criteria based on the objectives of the study to include/exclude the participants. The criteria include age, gender, stage of cancer, period of illness, and current medical condition of the participant. Participants were adolescents with cancer, coming from a children’s hospital during June and August 2022. Inclusion criteria were patients 8–18 years old with a cancer convalescence stage, with sufficient cognitive abilities to participate. Those patients with severe psychiatric illness or expressive disorders and below age were excluded. Both male and female participants and adolescents of different age groups and class sections were included in the sample to diversify the sample size.

### Rigor of study

To achieve the rigorous aim of the study, the most appropriate research design was employed based on the research’s stated objectives. As highlighted in the literature, data saturation has been achieved through careful sample size selection. A diverse sample has been ensured based on the gender and age group of participants. All ethical considerations have been considered while collecting data from the participants. Colaizzi’s 7-step analysis method was systematically performed^19^ to analyze, sort out, summarize, and extract the theme while analyzing the data. All these steps have ensured the rigor of this research.

### Data collection and analysis

In this study, semi-structured interviews were used to conduct in-depth interviews with the interviewees to understand and record the subjective experience of adolescents with cancer in rehabilitation. Then, I sorted out and analyzed data and important refined factors and discussed the relationship between various elements and the surrounding environment. The researcher determined the interview outline based on the literature review and nursing experts’ opinions, mainly including the following four questions: (1) What were your physical symptoms and psychological feelings during the treatment? (2) What changes have taken place in your family and social life during rehabilitation? (3) What are you worried about now? (4) What help do you need? To ensure the research meets all ethical guidelines for researching human participants, the study has followed the guidelines of four universal ethical principles^28^. These universal ethical principles are respect for persons, beneficence (minimizing risk), non-maleficence (intentional or unintentional harm due to the interview questions), and justice.

They recorded the interviewees’ interview content, facial expressions, and body language by recording and taking notes. The interview questions were shown to the participants in advance to ensure the participants were comfortable with the questions and that no intentional or unintentional harm came to the participants due to the interview questions. Moreover, the interview place was an independent, comfortable, and quiet room to provide the best environment for the participants to ensure their safety and comfort. During the interview, the researcher remained neutral, without any guidance or suggestion, paid attention to listening, questioning, and repetition, and appropriately questioned the conversation content of the research object, such as “Can you explain it in detail?” “Are there any other feelings?”. The interview was conducted for 30–40 min by two experienced researchers. The recorded materials will then be transcribed into written materials within 24 h after the interview and numbered according to the interview sequence. In the current study, Colaizzi’s 7-step analysis method was performed ^19^ to analyze, sort out, summarize, and extract the theme.

### Ethical considerations

Participation was voluntary, and written informed consent was obtained from participants’ parents or legal guardians to fulfill the respect for person obligation. The participant’s data was kept confidential, and no name or identity information was disclosed to protect the participants from any risk. Before the interview, the researcher explained the study’s purpose, significance, and interview procedure to the interviewees and their families, promised that the data collected would only be used for this study, kept their personal information strictly confidential, and obtained their informed written consent. Chongqing Medical University’s research ethical committee approved the study. a parent and/or legal guardian must have obtained informed written consent.

## Results

### Characteristics of participants

A total of 9 patients were interviewed. Table 1 shows the general and individual sociodemographic as well as the clinical characteristics of the patients.

**Table 1:** Individual characteristics of participants.

Participants	Age	Gender	Grade	Diagnosis	Treatment	Return to school
Participant1	9	G	Fourth	Ewing’s sarcoma	ABC	No
Participant2	8	B	Third	Lymphoma	AB	Yes
Participant3	17	G	High school	Ewing’s sarcoma	ABC	Yes
Participant4	8	B	Second	Non-Hodgkin’s lymphoma	ABC	No
Participant5	9	B	Fourth	Osteosarcoma	ABC	No
Participant6	13	G	Seventh	Acute lymphoid leukemia	B	No
Participant7	15	G	Ninth	Ewing’s sarcoma	ABC	No
Participant8	11	G	Fifth	Acute lymphoid leukemia	B	Yes
Participant9	14	B	Eighth	Non-Hodgkin’s lymphoma	ABC	Yes

Treatment: A operation; B radiotherapy; C chemotherapy.

### Perception of patients regarding the disease and its treatment

Through data analysis, the psychological course and emotional needs of adolescents with cancer in the convalescence stage were summarized into three themes: experience of adolescents with cancer, lack of emotions, and discomfort of returning to society.

#### Continuous negative emotions

Patients said their bodies had many side effects after many times of chemotherapy and radiotherapy. This had left a shadow in their hearts, and they would have painful emotions when they recalled it.

At that time, I vomited and repeatedly vomited. Once my hands were shaking when I vomited badly. I felt uncomfortable and wanted to vomit at the moment of thinking back to the time of treatment. (patient 1).

There is a very bad memory at that time. I had many symptoms, such as fever, pain, and swollen feet. Now my mother is very nervous if I have a fever. (patient 2)

I had a severe gastrointestinal reaction after taking the drugs. My stomach has been hurt after that time. Now when I think of taking the drugs, my stomach is still very uncomfortable. (patient 3).

I had a fever and vomiting. Anyway, it was very uncomfortable. I became very weak. Now I’m worried that it will be very serious again if I get a fever. Then I can’t cure it for a long time. (patient 6).

When asked what the patient was worried about, the patient expressed that they were concerned about the recurrence of the disease. Even though their treatment effect is good, most patients still worry about the change in their condition and the recurrence of the disease at any time.

(Bowing her head) I worried about recurrence; I had three operations and 12 chemotherapy before, then another two chemotherapy ensued, if it recurred, (…) (patient 1)

We have to come for a review every 6 months. Every review is very nervous. The Internet says that the recurrence rate of Ewing’s tumor is very high. (patient 3)

I’m worried about recurrence. If it recurs again, will I have to amputate like some people in the hospital? (patient 5)

Female patients suffered from hair loss during chemotherapy, and their external image changed. Shame in that period had an impact on their current behavior.

I am wearing a wig. It was given by volunteers when I went to the hospital for radiotherapy. It is made of real hair, and it’s very good. I don’t want to take it off even my hair grows. I think my hair must be different from before. (patient 6).

I don’t cut my hair. When it keeps growing, my hair has fallen out of the hat I have been wearing for the past 2 years. I don’t want to wear it. I will keep my hair. Keep more hair. When others look at my hair, I think I am bald.

I had to wear a hat in the past 2 years because my hair had fallen out. When people look at my head, I always think I’m bald without hair. Now I will not cut my hair; I will keep it long. (patient 7).

#### Emotionally intense behavior

When asked what the patients most want to do now, some proposed seeing their parents.

I want to see my parents (wiping away tears). They only stayed for a few days when they returned for the New Year. (…) I knew they were busy returning to the construction site to word, so I didn’t detain them. (patient 4).

I often call them, but no one answers. They are very busy. Then I don’t call them much anymore. (patient 5)

When asked if they wanted to return to school, most patients were willing to return to school. They almost lost contact with their classmates during the treatment.

After I got sick, my parents went out to work for money. Grandma took me to live with my aunt. After that, I haven’t seen any classmates, and we had no connection. I miss them very much. (patient 1).

I return to the original class; my good friends are friends. I don’t want to be downgraded, so my mother made up for my second-grade class. I was still doing my homework when I was in the hospital. (patient 2).

I don’t want to suspend school anymore. I don’t want to stay at home all day and go nowhere. I want to go to school and talk with teachers or classmates. (patient 3)

I want to go back to school (wiping away tears),(…) (patient 4)

#### Discomfort of returning to society

Some patients have stayed away from school for more than 1 year. After returning to school, they need to better adapt to life and study.

When is the holiday? I want to have a holiday soon (the school starts only 2 weeks during the interview). (patient 2)

I can’t keep up with the physique class in school. The teacher suggested to my mother that I suspend school for a while. (patient 3)

There is too much homework that I can’t finish it. Why is there so much homework? (patient 8)

My teachers and classmates always take special care of me because I am sick. I am not allowed to participate in some activities and games. In fact, I think I am not so weak. I want to join them. (patient 9).

Some patients are about to return to school. They are worried they cannot keep up with their studies and activities.

They have been studying for a year, and I am sure I can’t keep up with them when I go back. So I will have to go down one level. (patient 1)

I haven’t seen my teachers and classmates since I was ill, and I don’t know if they still remember me. (patient 4)

I think I have become stupid, and my health has deteriorated. When I go back to school, will those boys pull my hair? (patient 6)

## Discussion

The results of the present exploratory study illustrate the experiences and expectations of adolescents with cancer and identify barriers and facilitators for their mental and physical health and what should do by hospitals, schools, and society to improve patients’ affect, cognition, and behavior.

### Continuous Negative Emotions

Research shows that^22^ cancer experience significantly affects survivors’ long-term quality of life. Cancer pain and postoperative pain encountered in cancer experience will psychologically impact the growing children, and some survivors have post-traumatic stress disorder^23^. It has also been found^24^ that young cancer patients show negative emotional and psychological feelings after treatment and also gain posttraumatic growth. Consistent with the above research, this study found that the pain experienced during the treatment period will continue to the convalescence stage, leading to a continuing sense of psychological discomfort and hindering the establishment of self-confidence and self-esteem after returning to school. In addition, due to the fear of recurrence and the painful memory of treatment, patients have negative emotions such as anxiety and fear, which seriously reduce their quality of life.

It is suggested that medical staff pay more attention to the physical and psychological conditions of adolescents with cancer during hospitalization. First, doctors can relieve patients’ pain and discomfort through medical means. Second, nurses can correct patients’ misconceptions by improving communication and using psychological interventions to reduce negative emotions, such as relaxation, mindfulness training, and music therapy. Third, the Social Work Department in the hospital can organize various activities, such as drawing competitions, reading activities, etc. The above aims to improve the patient’s physical symptoms and psychological status, thus improving their long-term quality of life.

### Emotionally Intense Behavior

Adolescence is a critical stage of life’s psychological and social development, which is crucial in forming good habits, cultivating interpersonal skills, and establishing a healthy image^20,21^. In this period, suffering from major diseases and stagnation of campus life is a significant adverse event that may change life’s trajectory. Surgery, chemotherapy, radiotherapy, and targeted therapy are the primary means to treat children’s malignant tumors. The research results support that these treatments can cause temporary or permanent changes in adolescent psychology, making patients more sensitive to things in life^11^. More care should be given to the emotional needs of patients in the cancer convalescence stage^24,25^.

### Helping Patients Return to Society

Due to getting treatment in hospital, adolescents with cancer have been separated from school and society for a long time; they lost the company of classmates and friends^25^. They suffer from pain during treatment, their psychological resilience worsens, and their emotional needs for their families and peers become apparent. In this study, some patients’ parents went out to work to provide medical expenses. The patients lacked their parents’ companionship for a long time and gradually became sensitive and withdrawn. During in-depth interviews, they showed their missing and dependence on their parents but did not actively put forward their emotional needs to their parents. It is suggested that parents should improve communication with patients, such as increasing the frequency of telephone or video calls, paying more attention to their psychological state, and letting them feel more cared for.

In addition, we also found that the patient expected to return to school during the conversation. Returning to school is essential for a young patient who has recovered. It can help them return to everyday life and dramatically improve their mental health and social adaptability^26^. However, most patients lost contact and communication with their peers during treatment. Some patients received “special care” after returning to school, which did not bring them warmth but made them feel isolated, frustrated, and uncomfortable. This sense of isolation comes from the social exclusion perceived by the patients, which is also related to the lack of self-identity and the ability to identify the relationship between self and social groups in young patients^27^. Therefore, we can encourage patients to keep in touch with their peers during treatment and invite rehabilitation patients to share their mental journeys. During the convalescence stage, we can strengthen positive guidance through telephone follow-up or online community, listen to their existing ideas, observe their self cognitive, provide targeted psychological counseling, encourage them to join collective activities, and teach them to view others’ comments correctly. Considering the particularity of the disease, community medical staff should assess the patient’s physical condition and acting ability and provide personalized activity guidance. Teachers could guide patients to integrate into the collective quickly and arrange appropriate activities to enrich their campus life. Multidimensional social support can provide a buffer for patients under stress, help them adjust their mentality, enhance their self-confidence, make them more willing to contact society, and let them look forward to the future.

## Conclusions

After a long period of treatment, adolescents with cancer convalescence stage are sensitive and vulnerable. They are eager to return to family and society and do not want to be treated differently. During the treatment period, medical staff should provide more psychological support. So that patients can establish correct cognition and self-confidence, maintain communication with parents and peers, and make positive preparations for returning to society. At the same time, the organization should pay more attention to adolescents with cancer who are about to return or have returned to the community, build a sound and healthy support platform, promote the comprehensive physical, mental, and spiritual rehabilitation of patients, and improve their long-term quality of life eventually.

## Data Availability

The datasets used and/or analyzed during the current study are available from the corresponding author on reasonable request.

## References

[1] Catala-Lopez, F., Padron-Monedero, A., GBD. Adolescent Young Adult Cancer Collaborators Cancer (2022). The global burden of adolescent and young adult cancer in 2019: A systematic analysis for the Global Burden of Disease Study 2019. Lancet Oncol..

[2] Gupta S, Harper A, Ruan Y, Barr R, Frazier AL, Ferlay J, Steliarova-Foucher E, Fidler-Benaoudia MM (2020). International trends in the incidence of cancer among adolescents and young adults. J. Natl. Cancer Inst..

[3] Li HL, Wu S, Chan YC, Cheng SW, Guo W, Xiong J (2020). Early and mid-term mortality and morbidity of contemporary international endovascular treatment for type B aortic dissection - A systematic review and meta-analysis. Int. J. Cardiol..

[4] Bleyer A, Ferrari A, Whelan J, Barr RD (2017). Global assessment of cancer incidence and survival in adolescents and young adults. Pediatr. Blood Cancer..

[5] Quinn GP, Goncalves V, Sehovic I, Bowman ML, Reed DR (2015). Quality of life in adolescent and young adult cancer patients: A systematic review of the literature. Patient Relat. Outcome Meas..

[6] Lange M, Licaj I, Clarisse B, Humbert X, Grellard JM, Tron L, Joly F (2019). Cognitive complaints in cancer survivors and expectations for support: Results from a web-based survey. Cancer Med..

[7] Lange M, Joly F, Vardy J, Ahles T, Dubois M, Tron L, Winocur G, De Ruiter MB, Castel H (2019). Cancer-related cognitive impairment: An update on state of the art, detection, and management strategies in cancer survivors. Ann. Oncol..

[8] Janelsins MC, Kohli S, Mohile SG, Usuki K, Ahles TA, Morrow GR (2011). An update on cancer- and chemotherapy-related cognitive dysfunction: Current status. Semin. Oncol..

[9] Collins B, Mackenzie J, Tasca GA, Scherling C, Smith A (2014). Persistent cognitive changes in breast cancer patients 1 year following completion of chemotherapy. J. Int. Neuropsychol. Soc..

[10] Janelsins MC (2017). Cognitive complaints in survivors of breast cancer after chemotherapy compared with age-matched controls: An analysis from a nationwide, multicenter, prospective longitudinal study. J. Clin. Oncol..

[11] Dewar EO, Ahn C, Eraj S, Mahal BA, Sanford NN (2021). Psychological distress and cognition among long-term survivors of adolescent and young adult cancer in the USA. J. Cancer Surv..

[12] Epelman CL (2013). The adolescent and young adult with cancer: State of the art—Psychosocial aspects. Curr. Oncol. Rep..

[13] Ni X (2022). Socioeconomic inequalities in cancer incidence and access to health services among children and adolescents in China: A cross-sectional study. Lancet.

[14] Brower K, Schmitt-Boshnick M, Haener M, Wilks S, Soprovich A (2021). The use of patient-reported outcome measures in primary care: Applications, benefits and challenges. J. Patient-Rep. Outcomes..

[15] Aaronson NK (1993). The European organisation for research and treatment of cancer QLQ-C30: A quality-of-life instrument for use in international clinical trials in oncology. J. Natl. Cancer Inst..

[16] Force LM (2019). The global burden of childhood and adolescent cancer in 2017: An analysis of the Global Burden of Disease Study 2017. Lancet Oncol..

[17] Cai RQ, Liang LF, Zeng YL (2015). Psychological experience of adolescent malignant lymphoma patients: A qualitative research. Chin. J. Pract. Nurs..

[18] Liu XL, Lin J, Zhou CY (2010). Psychological experience of adolescent malignant patients with chemotherapy: A qualitative research. Med. Inf..

[19] Colaizzi P (1978). Psychological Research as the Phenomenologists Views it.

[20] Williamson H, Rumsey N (2017). Perspectives of health professionals on the psychosocial impact of an altered appearance among adolescents treated for cancer and how to improve appearance-related care. J. Psychosoc. Oncol..

[21] Wu WW, Chang JT, Tsai SY, Liang SY (2018). assessing self-concept as a mediator between anger and resilience in adolescents with cancer in Taiwan. Cancer Nurs..

[22] Tonsing KN, Ow R (2018). Quality of life, self-esteem, and future expectations of adolescent and young adult cancer survivors. Health Soc. Work..

[23] Rourke MT, Hobbie WL, Schwartz L, Kazak AE (2007). Posttraumatic stress disorder (PTSD) in young adult survivors of childhood cancer. Pediatr. Blood Cancer.

[24] Li Z, Zhang YP, Zhang Y (2020). Real experience of malignant bone tumor adolescents and young adults with amputation: Qualitative research. Chin. J. Modern Nurs..

[25] Al-Omari O, Wynaden D (2014). The psychosocial experience of adolescents with haematological malignancies in Jordan: An interpretive phenomenological analysis study. Sci. World J..

[26] Tremolada M, Taverna L, Bonichini S, Pillon M, Biffi A, Putti MC (2020). Pediatric patients treated for leukemia back to school: A mixed-method analysis of narratives about daily life and illness experience. Behav. Sci. (Basel)..

[27] Moore JB, Kaffenberger C, Goldberg P, Kyeung MH, Hudspeth R (2009). School reentry for children with cancer: perceptions of nurses, school personnel, and parents. J. Pediatr. Oncol. Nurs..

[28] Beauchamp T, Childress J (2001). Principles of Biomedical Ethics.

[29] Wilson A (2015). A guide to phenomenological research. Nurs. Stand..

